# Computer-Assisted In Sensu Exposure for Posttraumatic Stress Disorder: Development and Evaluation

**DOI:** 10.2196/mental.5697

**Published:** 2016-06-08

**Authors:** Nora Görg, Kathlen Priebe, Tilman Deuschel, Martin Schüller, Friederike Schriner, Nikolaus Kleindienst, Petra Ludäscher, Christian Schmahl, Martin Bohus

**Affiliations:** ^1^ Institute of Psychiatric and Psychosomatic Psychotherapy Central Institute of Mental Health Mannheim Medical Faculty Mannheim, Heidelberg University Mannheim Germany; ^2^ University of Applied Sciences Darmstadt Darmstadt Germany; ^3^ Jekyll & Hyde HQ Otzberg Germany; ^4^ Centre For Digital Media Vancouver, BC Canada; ^5^ Department of Psychosomatic Medicine and Psychotherapy Central Institute of Mental Health Mannheim Medical Faculty Mannheim, Heidelberg University Mannheim Germany; ^6^ Department of Psychiatry Schulich School of Medicine and Dentistry Western University London, ON Canada; ^7^ Faculty of Health University of Antwerp Antwerp Belgium

**Keywords:** dissociative disorders, behavior therapy, posttraumatic stress disorder, technology

## Abstract

**Background:**

Dissociative states during psychotherapy sessions reduce the benefit of exposure-based therapy for posttraumatic stress disorder (PTSD). Thus, in evidence-based therapeutic programs such as dialectical behavior therapy for PTSD (DBT-PTSD), therapists apply specific antidissociative skills to reduce dissociative features during *in sensu* exposure.

In addition to therapist-guided sessions, exposure protocols often require that the patients listen to audio recordings of exposure sessions in self-management. The problem of how to prevent dissociative features during such self-administered exposure exercises has not been resolved yet.

Hence, we developed the computer program MORPHEUS that supports the application of self-administered exposure exercises. MORPHEUS continuously monitors the level of dissociative states and offers state-related antidissociative skills.

**Objective:**

This study sought to examine the acceptance and feasibility of the MORPHEUS program.

**Methods:**

Patients who underwent 12 weeks of residential DBT-PTSD treatment used MORPHEUS during exposure exercises in self-management. After the treatment, they filled out evaluation questionnaires.

**Results:**

In sum, 26 patients receiving a 12-week standard DBT-PTSD program participated in this study; 2 participants could not be analyzed because of missing data. All the patients used MORPHEUS as often as it was required according to the DBT-PTSD treatment (2 to 5 times a week). The overall acceptance and feasibility as rated by the patients was high: for example, patients found the skills useful to block dissociation (mean 4.24 on a scale from 0 to 5, SD 0.24) and stated that they would use the program again (mean 4.72 on a scale from 0 to 5, SD 0.11). Furthermore, patients indicated that they would recommend MORPHEUS to a friend (mean 4.44 on a scale from 0 to 5, SD 0.12). In 82% (32/39) of the cases, the use of antidissociative skills was related to a decrease in dissociation. In 18% (5/39), dissociation remained unchanged or increased.

**Conclusions:**

The evaluative data suggest high acceptability and feasibility of MORPHEUS. Further studies should evaluate the effectiveness of the skills applied during the program.

**Trial Registration:**

World Health Organization International Clinical Trials Registry Platform: DRKS00006226; http://apps.who.int/trialsearch/Trial2.aspx?TrialID= DRKS00006226 (Archived by WebCite at http://www.webcitation.org/ 6hxuFbIUr)

## Introduction

*In sensu* exposure is a widely used and effective intervention in the treatment of posttraumatic stress disorder (PTSD) [1]. *In sensu* exposure protocols require patients to repeatedly imagine the traumatic incident with high emotional engagement [2-4]. Usually this is a bimodal process: therapist-guided sessions are followed by self-management sessions, during which the patients repeatedly listen to audio recordings of the therapist-guided exposure sessions. Such exposure exercises can be considered an emotionally demanding task in the absence of therapist support.

One problem that might arise during such exposure exercises is the occurrence of dissociative states. In their literature review, Craske and colleagues [5] conclude that the effects of exposure therapy are the result of learning new associations with traumatic memories. There is evidence that dissociative states during exposure impede emotional learning processes. Dissociation is described as an intermittent “disruption and/or discontinuity in the normal integration of consciousness, memory, identity, emotion, perception, body representation, motor control, and behavior” ([6] p291). High levels of dissociation in PTSD have been found in 15% to 33% of patients [7], which led to the inclusion of a dissociative subtype in the *Diagnostic and Statistical Manual of Mental Disorders, Fifth Edition* [6]. Patients show different levels of trait dissociation, but dissociation can also change on a state level as shown in an ambulatory assessment study in patients with a diagnosis of borderline personality disorder (BPD) or depression [8].

Dissociation positively correlates with the level of acute emotional distress and this association is highest in patients with BPD [9]. Dissociation can be provoked by imagining situations that previously led to dissociative states (interpersonal conflicts, trauma-related situations, and so on) [10,11]. This has strong implications for exposure-based interventions: retrieval of traumatic memories often induces high levels of emotional distress, which—mainly in individuals with high dissociation proneness—activates dissociative symptoms.

There is recent evidence showing that dissociation is associated with attenuated improvement after treatment of BPD [12] and other conditions such as obsessive-compulsive disorder [13,14]. In PTSD, the empirical evidence regarding the impact of trait dissociation on treatment outcome is relatively mixed [15-21]. However, a most recent analysis could show that state dissociation plays a powerful role in predicting negative treatment outcome [22].

Thus, therapists are faced with the challenge to apply exposure therapy in patients with high dissociation proneness. A range of strategies to cope with dissociation in PTSD can be found in the literature. In their Prolonged Exposure (PE) manual, Foa et al [23] provide treatment strategies for both emotional overengagement and state dissociation (eg, discriminating between the trauma itself and the traumatic memory, tactile stimuli such as holding the therapist’s hand). Several trauma-focused treatment approaches that are specifically tailored for PTSD patients with emotional dysregulation have included emotion regulation skills used in dialectical behavior therapy (DBT): DBT-PTSD [24,25], DBT+PE [26,27], and Skills Training in Affective and Interpersonal Regulation-Prolonged Exposure (STAIR-PE [15,28]. In skills-assisted exposure, which is an integral part of DBT-PTSD, emotion regulation and stress tolerance skills are used to find a balance between emotional re-activation of traumatic memories and the awareness of being in the present moment during exposure. In patients who tend to dissociate, stress tolerance skills such as cognitive or sensory distraction are applied to tackle state dissociation during exposure sessions [24,25].

To the best of our knowledge, no studies to date have tested the effectiveness of distraction to address state dissociation in patients with PTSD. However, the role of distraction tasks and the effect on working memory performance have been discussed as a potential treatment mechanism of Eye Movement Desensitization and Reprocessing (EMDR) therapy [29], for example, by Andrade et al [30]. In experimental studies, both the standard EMDR intervention of rapid eye movements and auditory distraction reduced the vividness and emotionality of negative autobiographical memories to a comparable extent [31]. Although a recent meta-analysis on dismantling studies suggests the use of eye movements in EMDR to be most effective [32], its specific benefit above other forms of distraction remains controversial [33]. In conclusion, distraction might downregulate distress during the processing of traumatic memories, which, in turn, might reduce the likelihood of dissociation.

A second consequence of *in sensu* exposure exercises in self-management is strong emotional activation. Patients with PTSD experience a range of trauma-related emotions such as shame, guilt, and anger, which correlate with treatment outcome and dropout rates in therapy [34-38]. DBT-PTSD aims at monitoring such specific trauma-related emotions to guide treatment decisions.

As stated above, most exposure-based treatments include exposure exercises in self-management. Typically, during these exposure exercises patients are requested to listen to audio recordings of the exposure sessions [23]. No therapist guidance is available during these emotionally demanding exercises. Computer programs and apps might have the potential to provide additional support during this challenging task.

So far, a few apps have been developed to support PTSD treatment. To our knowledge, only two of them have been published in clinical journals: PE Coach [39] and PTSD Coach [40]. Both have been developed by the US Department of Veterans Affairs and offer a feedback tool that tracks symptoms via the PTSD symptom checklist (PCL) [41].

PTSD Coach can be used as either a stand-alone tool or an adjunct tool to face-to-face-therapy [42]. Coping strategies (eg, behavioral activation) are offered according to the patient’s subjective units of distress (SUDs). Furthermore, the app offers a range of sensory grounding techniques (eg, “Focus all of your attention on the feel of a pebble or coin in your hand.”) in case patients report dissociative symptoms. Users reported high levels of satisfaction and perceived helpfulness of the app [40,43]. However, a pilot randomized controlled trial on the effectiveness of PTSD Coach did not find a statistically significant reduction of PTSD symptoms in comparison with a waitlist control group [44]. PE Coach on the other hand is an adjunct to PE therapy, which enables patients to record and listen to their exposure sessions. The app monitors adherence to the exposure exercises and SUDs before and after each exercise. High satisfaction with the app was reported in a longitudinal case study with two soldiers [45].

Although these apps appear to be promising, they do not allow monitoring dissociation while listening to the recording. Furthermore, PE Coach does not provide strategies to cope with state dissociation. Moreover, apart from recording SUDs, none of the apps provides tools for monitoring other trauma-associated emotions. Therefore, we developed a computer program (MORPHEUS) that is designed to support self-administered *in sensu* exposure. It monitors state dissociation and offers skills to tackle dissociation if necessary. Furthermore, MORPHEUS is used for the feedback and monitoring of specific trauma-related emotional experiences. The study outlined here aimed at examining the acceptance and feasibility of the MORPHEUS program.

## Methods

### The MORPHEUS Program

MORPHEUS is a computer program that allows computer-assisted *in sensu* exposure exercises in self-management during the treatment of PTSD (see Treatment section). It can be used to listen to recordings of *in sensu* exposure sessions. A video showing the main functions of the program can be found in Multimedia Appendix 1.

Usability was a key requirement for MORPHEUS. The program was developed based on requirements expressed in interviews with patients and therapists at the PTSD unit of the Central Institute of Mental Health (CIMH), Mannheim (Germany). MORPHEUS was iteratively improved based on users’ feedback.

#### Audio Recording

**Figure 1:**
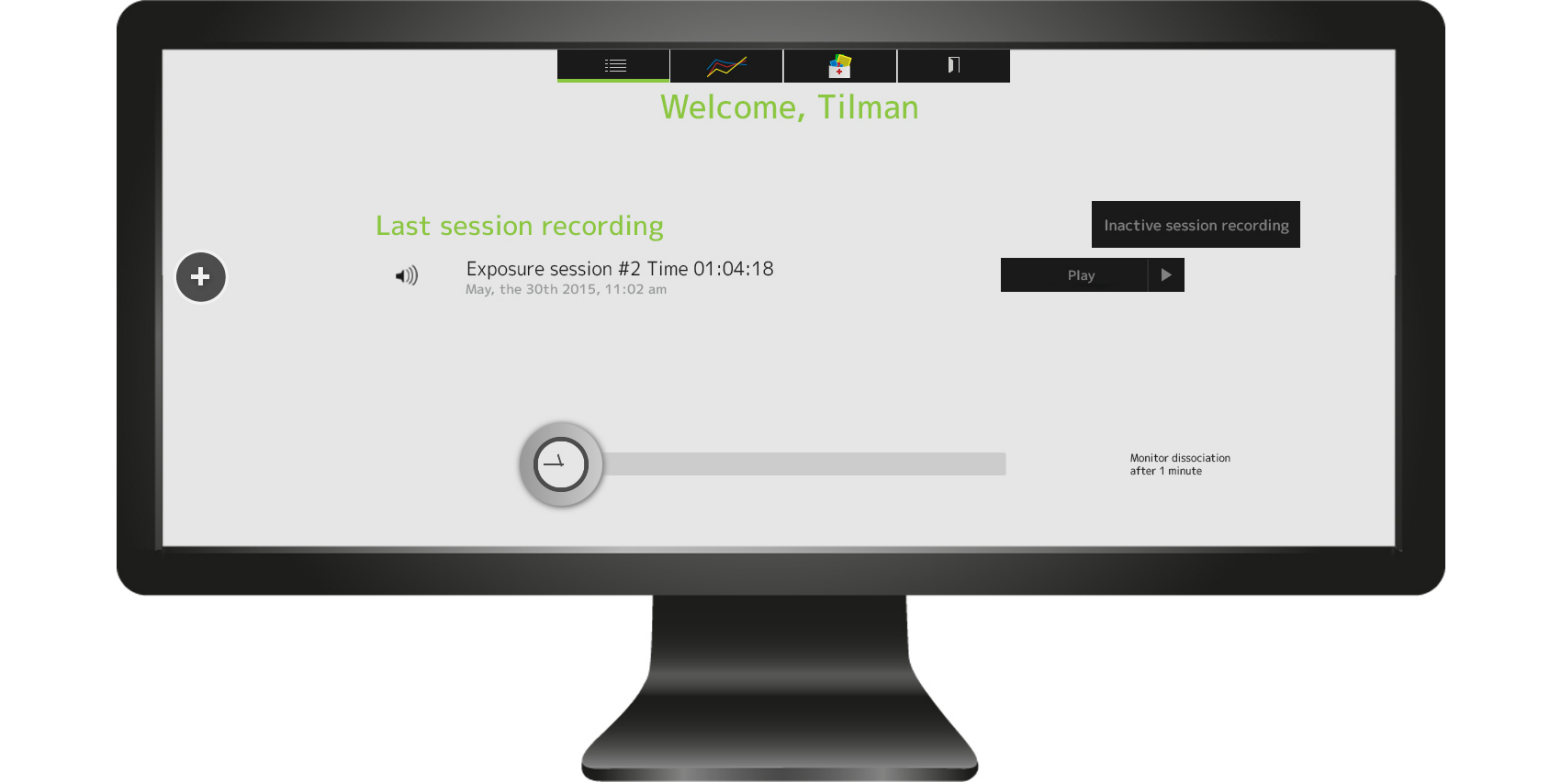
Main screen: selection of audio recording and time interval for the iterative question for state dissociation.

**Figure 2:**
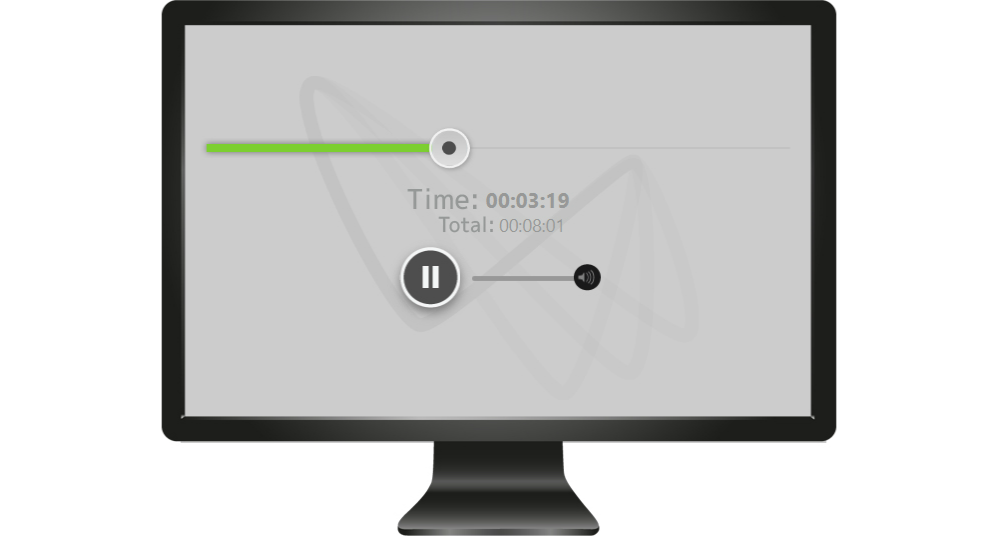
Session recording with MORPHEUS.

Patients record their exposure sessions on a digital voice recorder where MORPHEUS is preinstalled. When the voice recorder is connected to a computer, the program imports and plays the recordings automatically (Figures 1 and 2).

#### Monitoring of State Dissociation and Skills

While playing the recorded session, patients can monitor the level of state dissociation on a scale from 0 (not at all) to 100 (very much) in self-adjusted time intervals between 1 and 15 minutes. The question for state dissociation can be deactivated as well. During therapy, patients are trained to evaluate and monitor their individual levels of state dissociation on this scale. This intervention aims at increasing the patient’s awareness of dissociation and thus fostering their ability to address these symptoms.

A dissociation level of 70 is defined as an anchor point: at that point, cognitive functioning is severely impaired, and sensory awareness of the here and now is perceived as increasingly “unreal.” This concept corresponds to the idea of high aversive distress as defined by DBT [46]. According to DBT, high aversive distress requires stress tolerance skills that aim at the immediate downregulation of distress, for example, via distraction. Such distraction skills are used in MORPHEUS to block dissociative states. The program does not offer any other skills from DBT.

**Figure 3:**
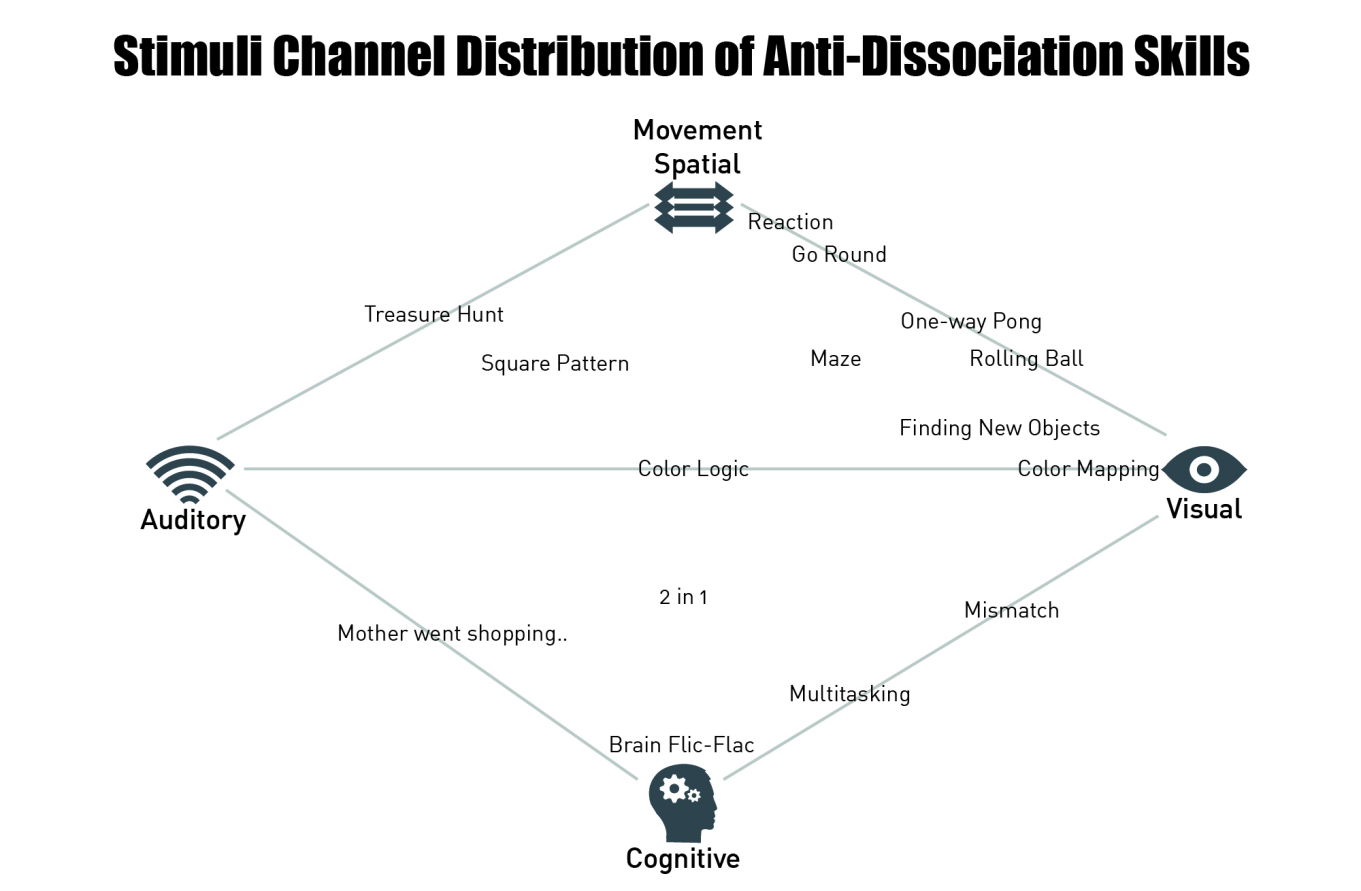
Distribution of the 15 MORPHEUS skills along the 4 sensory domains.

**Figure 4:**
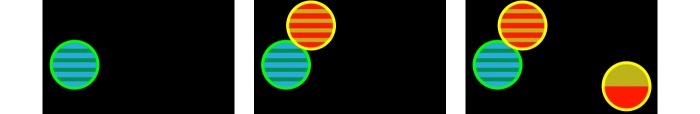
Finding new objects: in this skill patients need to click on the new object appearing on the screen.

If the patient endorses a dissociation rating of higher than 70, MORPHEUS randomly offers one of 15 available skills. These skills can be either used immediately or skipped. Patients can also predefine a subset of favorite skills from which the program chooses to offer one. On-demand use of skills is possible at any time during the exposure exercises and skills are available at any time after the exposure exercise as well. Patients can access and practice skills within the “skills box” of the program. Skills require a time frame of approximately 1 minute each or can be stopped earlier. Once it ends, MORPHEUS again asks for the level of dissociation. Skills in MORPHEUS are quick digital tasks. These tasks require full attention and serve as distractors that interfere with dissociation. MORPHEUS applies skills that address 4 sensory domains: spatial, cognitive, visual, and auditory (see Figures 3 and 4 and Table 1).

If a user is inactive for more than 10 seconds despite being asked for input (eg, during a skill or the monitoring question), white noise and an unpleasant tone (1-kHz sinus wave) are presented and the screen background alternates from white to black. This strong sensory signal serves as a distractor that helps the patient to interrupt a pronounced dissociative state and to reorient to the present.

**Table 1:** Overview of the skills in MORPHEUS.

Skill name	Task	Sensory domains
Treasure hunt	Find an audio signal with a noise detector.	Auditory Spatial
Square pattern	Click if you see a square pattern or if you hear a sound.	Visual Cognitive Auditory
Color logic	Click on the announced color.	Visual Auditory Cognitive
Color mapping	Click on the square that matches the background color.	Visual Auditory Cognitive
Go round	Don't let the moving squares touch the cursor.	Spatial Visual
Maze	Remember the luminescent squares on a maze and then click on them with the cursor.	Visual Cognitive Spatial
One-way pong	Catch a moving ball.	Visual Spatial
Rolling ball	Navigate a ball down the screen.	Visual Spatial
Reaction	Move the cursor to the objects that appear as soon as possible.	Visual Spatial
Finding new objects	Click on each new symbol.	Visual Cognitive
Mismatch	Find the symbol that differs from the rest.	Visual Cognitive
Brain Flic-Flac	Solve an arithmetic task.	Cognitive
Multitasking	Solve an arithmetic task and choose a symbol that differs from the others.	Cognitive Visual
2 in 1	Click on the symbol if it matches one of two symbols displayed above or if you hear a sound.	Cognitive Visual Auditory
Mother went shopping	Play the sounds that you've just heard.	Cognitive Auditory

#### Monitoring and Feedback of Emotions

**Figure 5:**
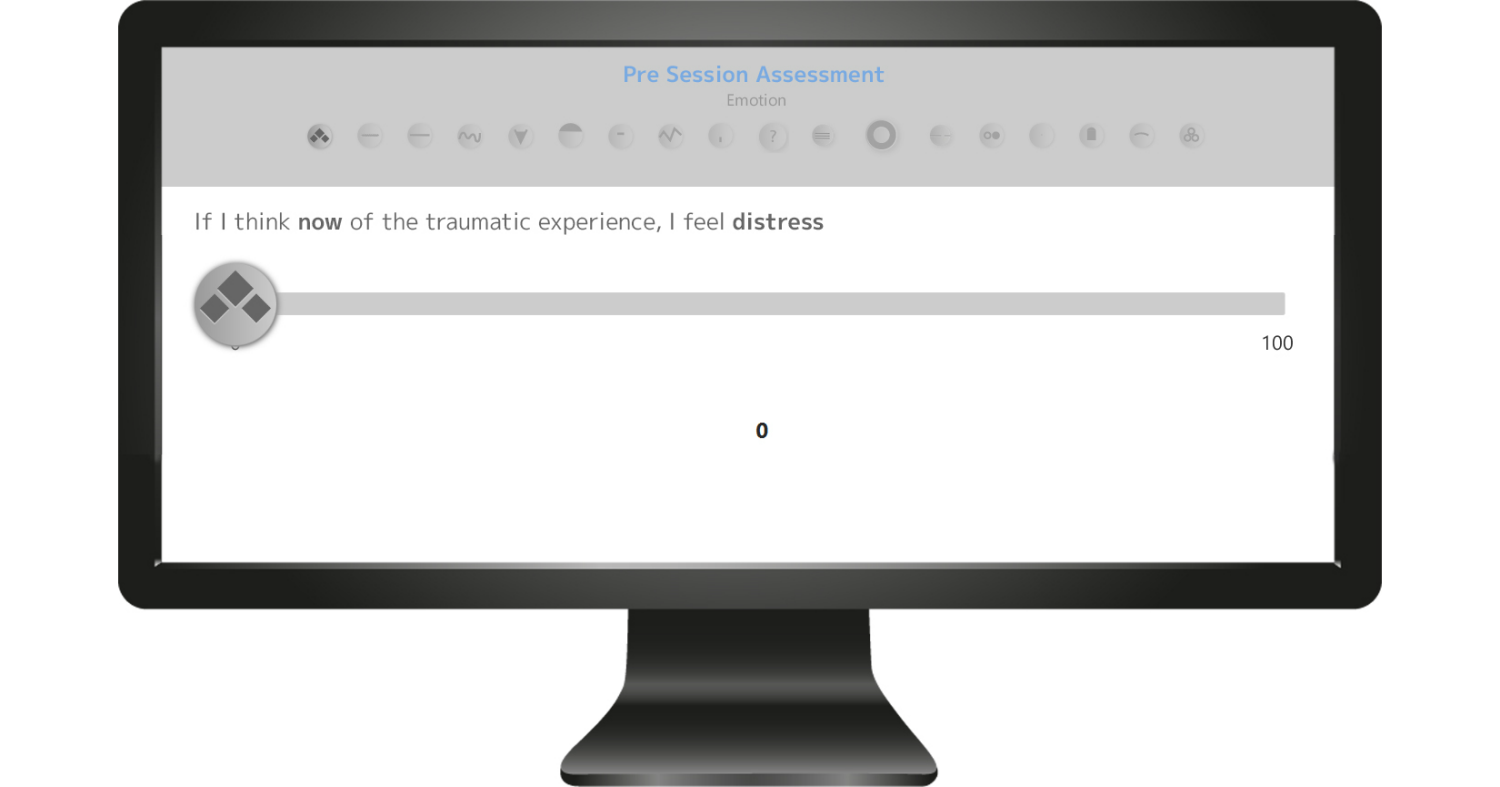
Pre- and postsession assessments of dissociation (Dissociation Tension Scale-4) [8] and different trauma-related emotions. The questions on emotions adopt the following pattern: “If I think of the traumatic experience now, I feel [emotion, e.g. distress].”.

**Figure 6:**
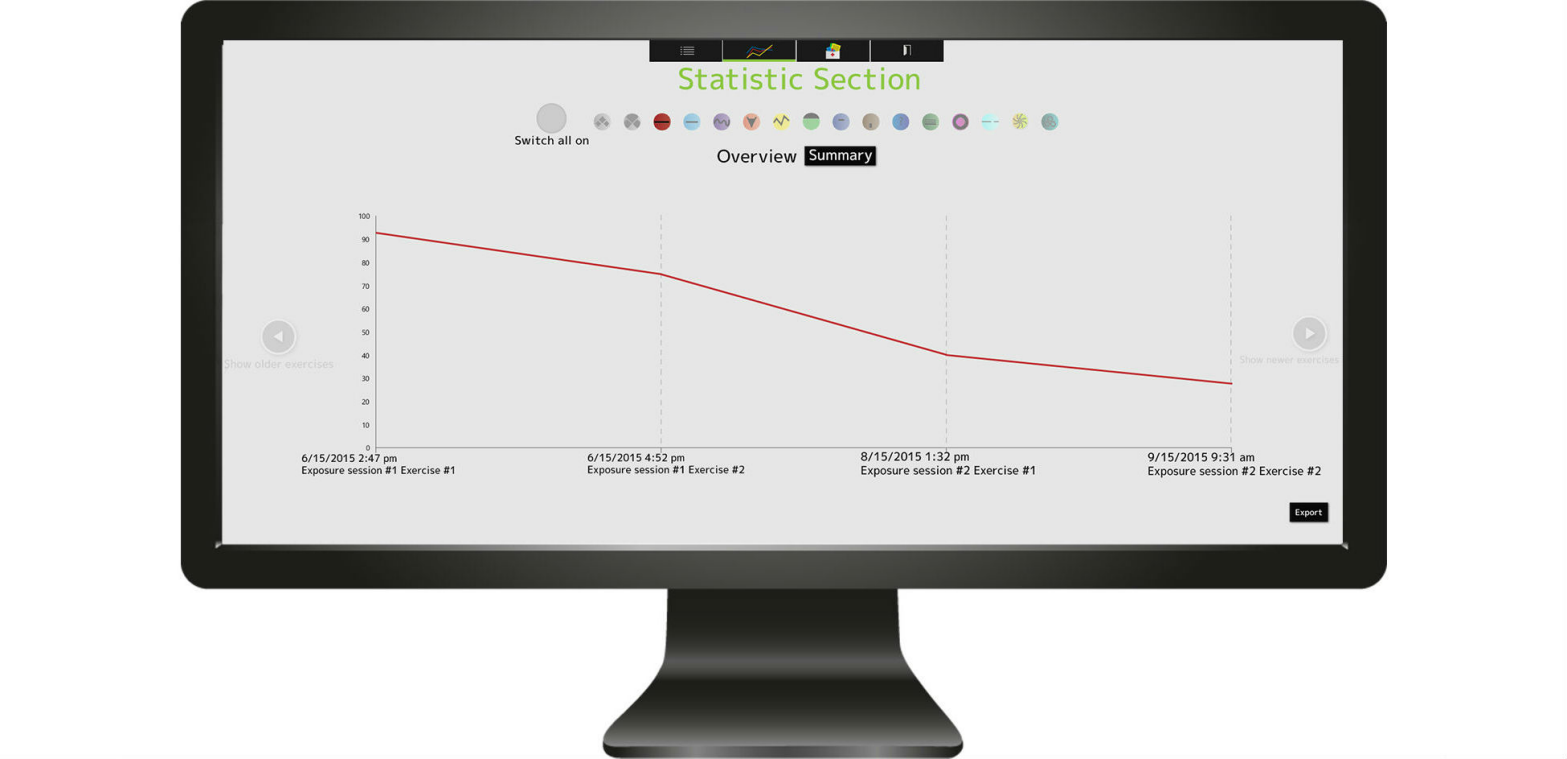
Statistics overview of trauma-related emotions and experiences. In this case, the patient chose to monitor fear (red). The time-dependent change of trauma-related emotions can be exported to a CSV file for research and feedback purposes.

Several items are rated on a scale from 0 to 100 before and after each exposure session: the intensity of trauma-related emotions (guilt, shame, fear, anger, and helplessness), distress, dissociation, and acceptance of the traumatic event as part of the personal history (see Figure 5). State dissociation is measured with the Dissociation Tension Scale-4 (DSS-4) [8] with 4 questions on distorted hearing, derealization, depersonalization, and analgesia. The course of these trauma-related emotions and experiences is visualized within the statistics section of the program (see Figure 6). Data on trauma-related emotions in MORPHEUS and during exposure sessions will be published elsewhere.

### Study Design

The participants received 12 weeks of residential DBT-PTSD at the CIMH in Mannheim, Germany, where MORPHEUS is a part of the standard treatment protocol. After 3 weeks in treatment, the participants were informed about the study and given a short introduction to the program by a doctoral level student (NG). Technical support was provided if any problems arose with MORPHEUS or with the devices needed to run the program (laptop, audio-recording device). If informed consent was given, the patients were directly asked to fill out a questionnaire on demographic details. The evaluation questionnaire was filled out after the exposure phase. The study has been approved by the Ethics Committee of the Medical Faculty Mannheim, Heidelberg University, and conducted according to the Declaration of Helsinki. The trial was registered in the World Health Organization International Clinical Trials Registry Platform (DRKS00006226).

### Treatment

In this study, MORPHEUS was used as part of a DBT-PTSD residential treatment [24,25]. The patients received a 12-week multicomponent residential treatment. DBT-PTSD is based on principles of DBT and comprises trauma-specific cognitive interventions, exposure, as well as compassion-focused interventions. Exposure sessions usually take place between the fifth and the tenth week of treatment. If more than one trauma had been experienced, patients select the currently most distressing event as index trauma. During *in sensu* exposure, patients are asked to imagine and report the index trauma. In order to tackle state dissociation, stress tolerance skills (eg, distraction) are applied during exposure when necessary. In addition to the therapy sessions, patients were requested to listen to audio recordings of the exposure sessions, applying MORPHEUS on weekdays with no therapy session, that is, 2 to 5 times a week.

### Participants

**Figure 7:**
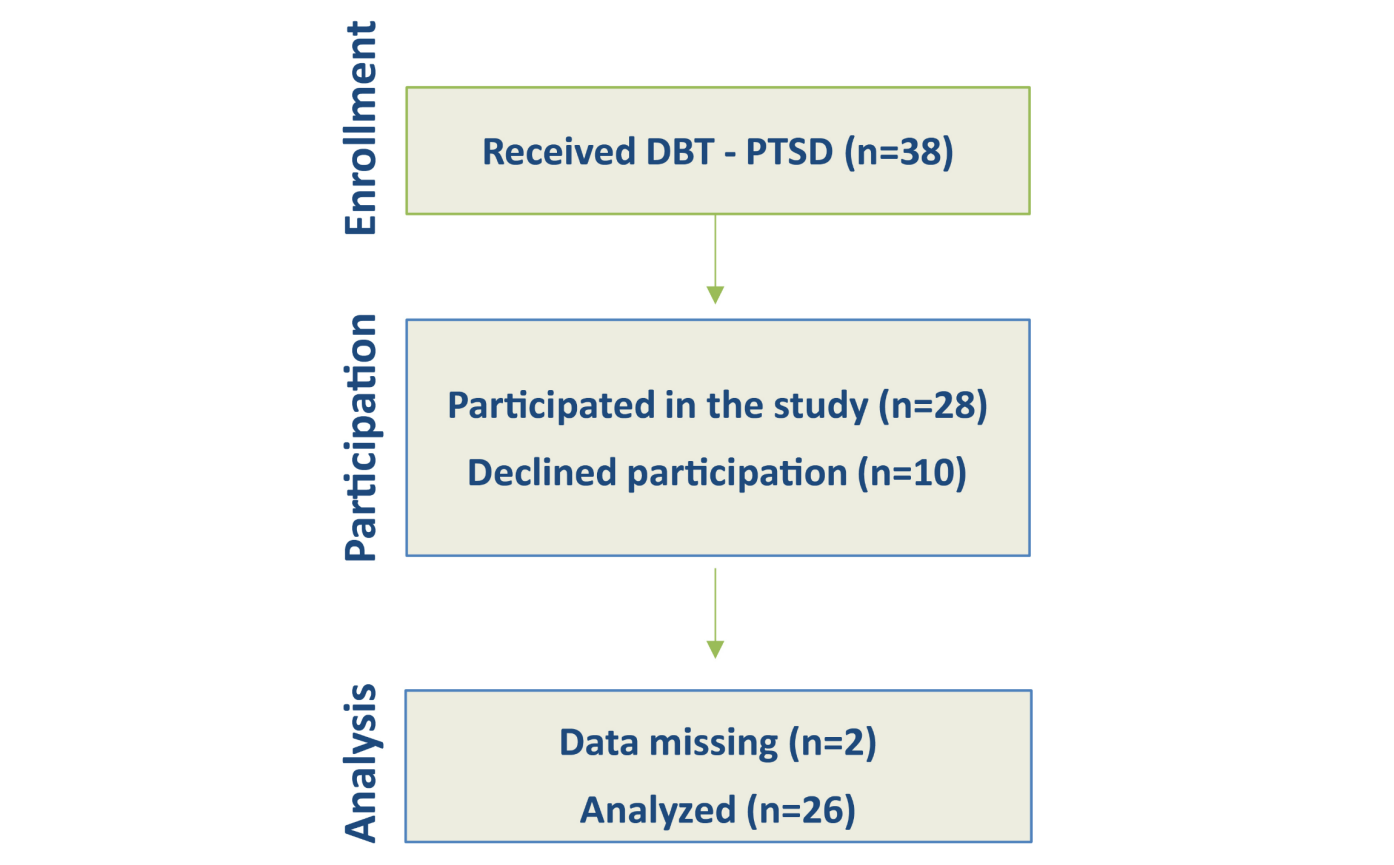
Patient flowchart. DBT-PTSD: dialectical behavior therapy for posttraumatic stress disorder.

The intake criteria for this study were as follows: (1) PTSD according to the *Diagnostic and Statistical Manual of Mental Disorders, Fourth Edition, Text Revision* (*DSM-IV-TR* [47]); and (2) residential DBT-PTSD treatment within the PTSD unit of the CIMH. Exclusion criteria included (1) acute drug intake and (2) medical contraindications to exposure-based treatment (eg, body mass index less than 16, cardiovascular disease). During the study period from July 2014 to July 2015, a total of 38 patients received regular treatment at the PTSD unit of the CIMH. Ten patients declined participation and 2 evaluation questionnaires were not returned. Thus, a final sample consists of 26 patients (25 females, 1 male; see Figure 7). Participants had a mean age of 42.15 years (SD 9.37). On average, they had received 10 years of education (SD 1.02). The average intake score of state dissociation during the session as measured with the DSS-4 was 3.05 out of 9 (SD 2.32).

As shown in Table 2, most of the patients had experienced childhood physical or sexual abuse. Other trauma categories included physical or sexual violence during adulthood, accidents, imprisonment, and suicide or violent death of a family member. Treatment focused on exposure to sexual or physical abuse experiences for all the patients.

**Table 2:** Co-occurring Axis I disorder and trauma categories (N=26).

Sample Characteristics	n
**Current comorbidity:**	
Major depression	20
Dysthymia	1
Eating disorders	5
Panic disorder	3
Specific phobia	3
OCD^a^	2
GAD^b^	1
Substance abuse	1
Somatization disorder	1
**Trauma category:**	
Sexual abuse after age of 18 years	9
Sexual abuse before age of 18 years	23
Physical abuse after age of 18 years	8
Physical abuse before age of 18 years	13
Accident	2
Imprisonment	1
Suicide or violent death of family member	2

^a^OCD: obsessive-compulsive disorder.

^b^GAD: generalized anxiety disorder.

### Assessments

Before admission, patients were interviewed with the German version of the Structured Clinical Interview for *DSM-IV-TR* (SCID-I) [48] to check for the inclusion criterion (PTSD) and co-occurring disorders. Additionally, patients were interviewed based on the BPD section of the International Personality Disorder Examination [49], which is part of the standard diagnostic procedure for the CIMH PTSD unit. The interviewers were graduate-level psychologists with ongoing training in cognitive behavioral therapy and supervised by senior-level clinicians.

**Figure 8:**
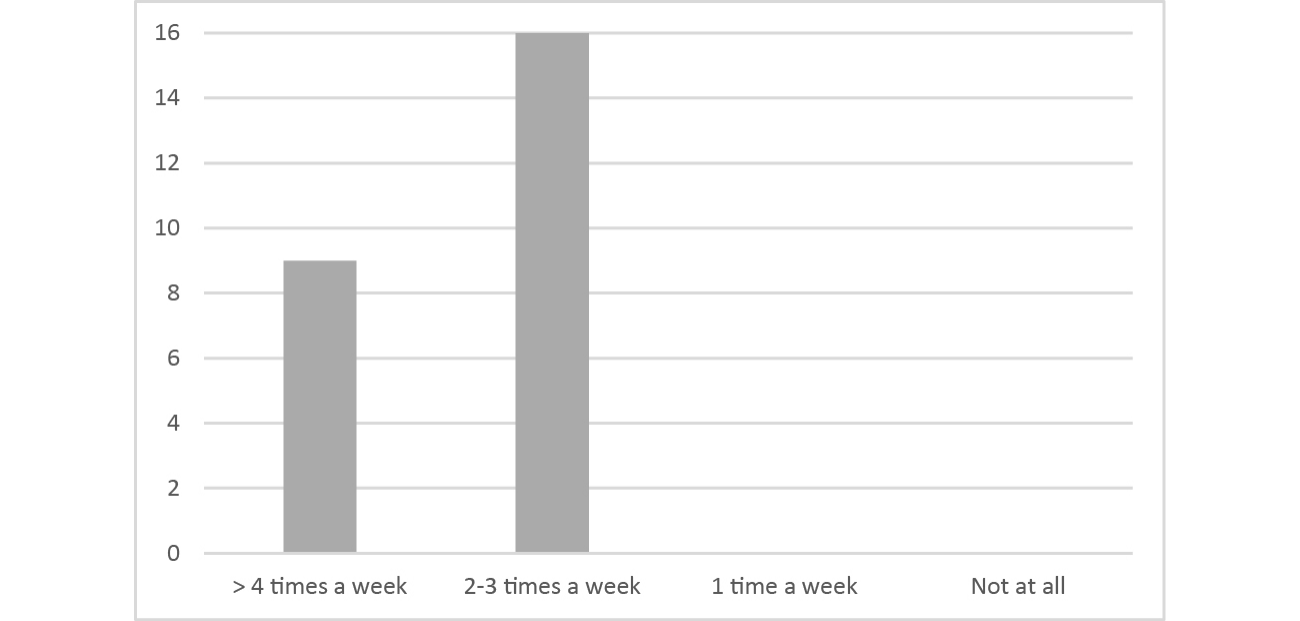
Frequency of MORPHEUS use per week on a 4-point Likert scale (N=25).

**Figure 9:**
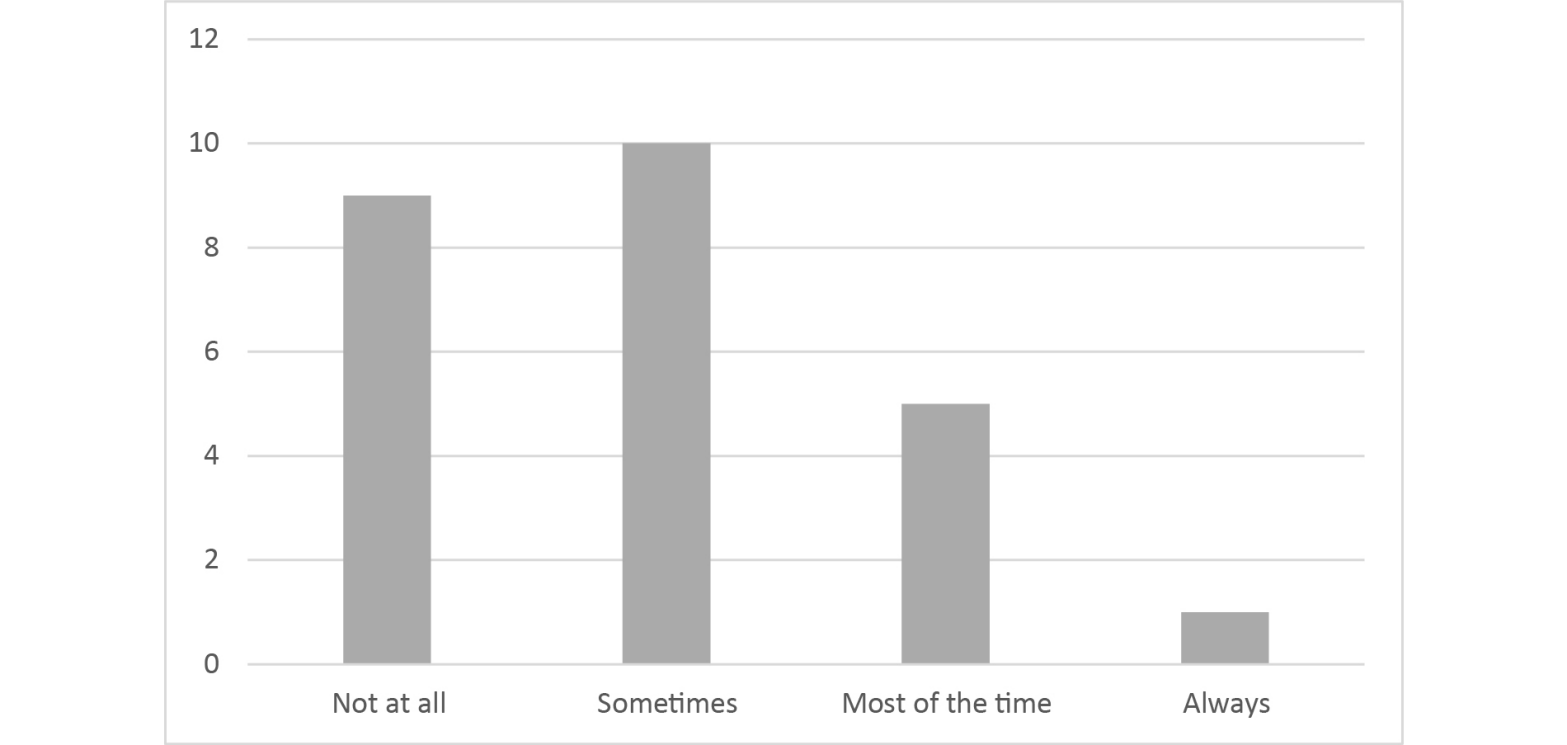
Frequency of digital skill use (N=25).

**Figure 10:**
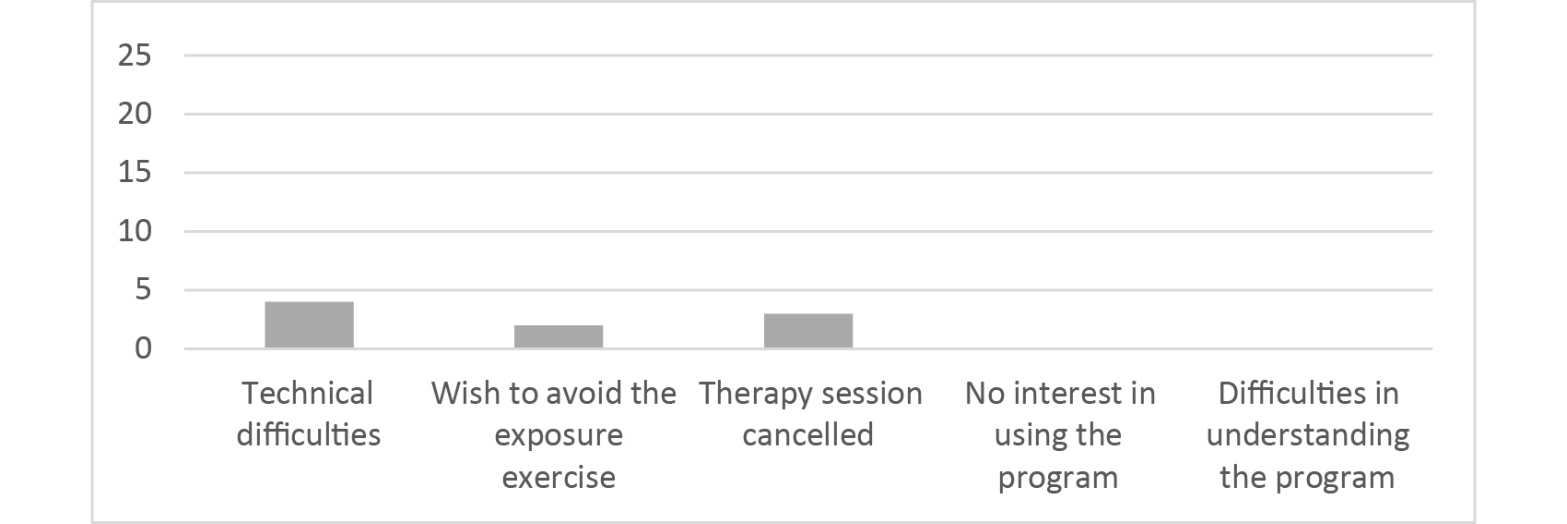
Reasons for not using MORPHEUS (N=9).

Diagnoses were double-checked by experienced clinicians at admission to the PTSD unit. The acceptance of MORPHEUS was assessed with a 17-item questionnaire (see Figures 8,9, and 10 and Table 3). The questionnaire measures overall satisfaction with the program and with the digital skills, usability, as well as perceived helpfulness on a 6-point Likert scale ranging from 0 to 5 (Table 3). Frequency of skills use within MORPHEUS was rated on a 4-point scale (Figure 9). In addition, patients were asked in an open-format question what was helpful, whether they had suggestions for improvement, and what kind of additional support during MORPHEUS use they would have liked to see.

### Data Analysis

All data were analyzed on a descriptive level. Subjective ratings of dissociation on a scale from 0 to 100 were compared before and after skills use. As to the qualitative data, patients’ comments were analyzed using procedures adapted from content analysis according to Mayring [50].

## Results

All patients received *in sensu* exposure and were supposed to use the MORPHEUS program during exposure exercises. Mean duration of the exposure phase was 4.3 weeks (SD 1.9).

### Quantitative Data

All patients used the program as often as it was required in the standard DBT-PTSD protocol, that is, at least 2 to 5 times a week (see Figure 8). As can be seen in Figure 9, most patients used the skills in MORPHEUS at least sometimes (16/25, 64%), whereas 9/25 patients (36%) never used them and 1 patient did not answer that question.

According to our session logs, a total of 10 patients used antidissociative skills. Because of technical difficulties, data were not available for 5 patients. On average, these patients started the skills 3.9 times. In 82% of the cases (32/39), the use of antidissociative skills was related to a decrease in dissociation. In 5%, (2/39) dissociation remained unchanged, and in 13%, (5/39) the scores of dissociation increased. In these outlier cases, the skills might have elicited great distress for the patients, maybe because they were not able to concentrate or because they felt enormous pressure to perform well in the skills. Mean change of pre- to post-skill dissociation was 17.82 with a standard deviation of 29.65.

Altogether, 96% (24/25) of the patients reported that they used other DBT skills, apart from the digital skills in MORPHEUS, during exposure (see Table 3), and 1 patient did not answer that question. Some participants (n=9) named reasons for not using the program (multiple choices were possible), such as technical difficulties (n=4), the wish to avoid exposure sessions (n=2), and the cancellation of treatment sessions (n=3; see Figure 10).

The results of the evaluation questionnaire revealed an overall high acceptance of the program as perceived by the patients (see Table 3). In the following, percentages are reported for patients who indicated at least a moderate agreement with the statements in the questionnaire, that is, who rated at least a 3 on this scale from 0 to 5. Skills were rated as highly useful to block dissociation: 95% (20/21) of the patients rated the skills at least moderately useful to block dissociation (mean 4.24, SD 0.24; n=21). In all, 100% (25/25) of the patients who answered the item indicated at least moderate agreement to the statement that they would recommend the program to a friend (mean 4.44, SD 0.12; n=25). Furthermore, 96% (25/26) of the patients rated the program at least moderately helpful (mean 4.62, SD 0.15; n=26). Altogether, 100% (25/25) of the patients who answered that question expressed at least a moderate level of perceived control during the use of MORPHEUS (mean 4.2, SD 0.15; n=25).

**Table 3:** Acceptance of MORPHEUS: percentages of patients who indicated a rating between 0 and 5 on the Likert scale (N=26).

Question		0	1	2	3	4	5	Mean (SEM)	Missing, n
**Program in general**									
	During the use of the program I had 0: no control at all - 5: complete control	0% (0/25)	0% (0/25)	0% (0/25)	20% (5/25)	40% (10/25)	40% (10/25)	4.20 (0.15)	1
	The program meets my expectations 0: not at all - 5: very much	0% (0/24)	0% (0/24)	0% (0/24)	8% (2/24)	50% (12/24)	42% (10/24)	4.33 (0.13)	2
	I did get along with the program 0: not at all - 5: very well	0% (0/26)	0% (0/26)	0% (0/26)	12% (3/26)	12% (3/26)	77% (20/26)	4.65 (0.14)	0
	I found the program 0: not at all helpful - 5: very helpful	0% (0/26)	0% (0/26)	4% (1/26)	4% (1/26)	19% (5/26)	73% (19/26)	4.62 (0.15)	0
	I would recommend the program to a friend 0: not at all - 5: very much	0% (0/25)	0% (0/25)	0% (0/25)	4% (1/25)	48% (12/25)	48% (12/25)	4.44 (0.12)	1
	I would 0: not like to use the program - 5: like to use the program during therapy	0% (0/25)	0% (0/25)	0% (0/25)	4% (1/25)	20% (5/25)	76% (19/25)	4.72 (0.11)	1
	The support I got for using MORPHEUS was 0: not sufficient at all - 5: absolutely sufficient	0% (0/26)	0% (0/26)	0% (0/26)	4% (1/26)	12% (3/26)	85% (22/26)	4.81 (0.10)	0
**Skills**									
	The skills were 0: very unuseful - 5: very useful in order to avoid dissociation	0% (0/21)	5% (1/21)	0% (0/21)	19% (4/21)	19% (4/21)	57% (12/21)	4.24 (0.24)	5
	I was 0: very unsatisfied - 5: very satisfied with the digital skills in MORPHEUS	0% (0/19)	5% (1/19)	0% (0/19)	21% (4/19)	47% (9/19)	26% (5/19)	3.89 (0.23)	7
	The digital skills in MORPHEUS 0: didn't work as I would like to - 5: worked exactly the way I liked to	0% (0/19)	5% (1/19)	11% (2/19)	11% (2/19)	53% (10/19)	21% (4/19)	3.74 (0.25)	7
	During my exercises with MORPHEUS I used my own skills beyond the skills in the program 0: never - 5: very often	4% (1/25)	0% (0/25)	8% (2/25)	16% (4/25)	28% (7/25)	44% (11/25)	3.96 (0.26)	1

^a^SEM: standard error of the mean.

### Qualitative Results

Altogether, 23 patients reported what they found helpful within the program in an open question format: the answers were related to the skills (eg, “no dissociation possible, skills directly available”), easy handling (eg, “suitable for people who haven’t worked with computers before”), dissociation monitoring question, the possibility to pause recordings, and the graphs about the longitudinal course of trauma-related experiences and emotions (eg, “You were able to see any time what you have reached in your therapy”). One patient referred to the intervention as such (“hearing how my voice got stronger and how I acted opposite”).

In all, 12 patients suggested ways to improve the program, again relating to the skills (“the program should offer skills to differentiate between present and past, for example: “Where am?” “How old am I?”), the pre- and postsession assessments (“the screen with the question disappeared too quickly after logging in the answer”), and the upload of the recording (“ability to upload two recordings with only one pre- and post-exercise assessment”).

Only 3 patients answered the question whether they had wished additional information. One patient indicated confusion about the pre- and post-session ratings of trauma-related emotions (“How do I rate the questions if two trauma networks overlap? If there are no feelings, what should the rating be?”). Another participant mentioned problems regarding asking for help while using the program (“I didn’t have the heart to ask for additional support”). A third patient expressed the wish for more support during the first use (“When you do your first exposure exercise with the program, someone should be with you, because I couldn’t cope with MORPHEUS the first two times”).

## Discussion

### Principal Findings

Our preliminary data show high acceptance and satisfaction with the computer program MORPHEUS as a self-administered adjunct to therapist-guided exposure therapy. Patients used the program as often as it is recommended in DBT-PTSD. Overall, patients found the skills helpful to block dissociation, and they stated that they would use the program again in therapy and would recommend it to a friend. In an open question format, patients mentioned all the elements within the program as being helpful. However, patients often used their own skills rather than the digital skills. Suggestions for improvement related to the skills, upload of session recordings, and assessment questions. Use of antidissociative skills by the participants was usually related to a decrease in dissociation (82%). Because of the lack of a control group and the small sample size, this result should be considered as preliminary and requires replication from a study designed to establish efficacy of MORPHEUS.

### Comparison With Prior Work

To our knowledge, this is the first study testing the longitudinal use of technology to reduce dissociative states and track trauma-related emotions during exposure exercises in PTSD therapy. Other programs such as PE Coach and PTSD Coach emphasize other aspects of PTSD therapy such as breathing retraining, in vivo exercises, and psychoeducation. Acceptance rates of MORPHEUS were comparable to the ratings from the feasibility study of PTSD Coach, using veterans as participants [40]. Thus, the usability of our program seems comparable to existing technology in PTSD treatment.

### Limitations

On the basis of the finding that patients more often used the skills already learned in DBT skills groups as compared with the MORPHEUS program, it can be assumed that patients rely heavily on already established antidissociative skills that are available without electronic devices. However, we did not test whether monitoring of dissociation in MORPHEUS might have increased patient’s awareness of state dissociation. Increased awareness of dissociation might have prompted the use of skills outside of MORPHEUS. Future versions of this program should prompt skills use outside of MORPHEUS in addition to the digital skills, comparable to the grounding techniques suggested in PTSD Coach.

At this point in our research project, we cannot provide data about the efficacy of the digital skills in MORPHEUS to block dissociation. To the best of our knowledge, studies that empirically test the efficacy of distraction skills or grounding techniques to block dissociation during trauma exposure do not exist. Thus, future studies will test whether the skills in MORPHEUS are effective in blocking dissociation during exposure as compared with a control condition without skills.

The program was explained to the patients by one of the authors (NG) during a 30-minute information session. This procedure might have resulted in a bias within the evaluation questionnaires toward more positive ratings. All patients learned about digital antidissociative skills within this session and also had the chance to test some of the skills. This might explain why patients gave overall positive ratings for the skills while at the same time indicating infrequent use of the MORPHEUS skills during the actual exposure exercises. During the trial period, one patient spontaneously reported that she used the MORPHEUS skills after the exposure exercises in order to reduce dissociative states. Thus, she might have rated the skills as highly useful to block dissociation yet at the same time she did not use them during the exposure. However, only skills use during the exposure exercise was monitored for the purpose of this study.

Some technical problems arose because of the need to use different electronic devices to run the MORPHEUS program (computer, digital voice recorder). Therefore, a mobile app version of MORPHEUS was developed and a prototype with the MORPHEUS skills is freely available for Android devices [51].

A major limitation of this study is that all except one participant were female. The PTSD unit of the CIMH is specializing in the treatment of consequences of early interpersonal violence. However, the majority of patients seeking this treatment are female (around 11 out of 13). Studies on the prevalence of childhood sexual abuse point to the direction of a higher prevalence of severe forms of victimization in girls than in boys (eg, higher rate of penetration; see [52-54]). As a consequence, the population of treatment seekers is represented sufficiently by our sample. However, future studies should be more adept to recruit male patients and patients with different types of traumatic experiences. In addition, the program should be tested within outpatient samples.

### Conclusions

With MORPHEUS, patients can record and listen to the therapy session and check the feedback of pre- and postexercise assessments. Our data suggest high acceptability and feasibility of the program. Further studies will evaluate the effectiveness of using MORPHEUS through mobile apps.

## Appendix

Multimedia Appendix 1 Presentation and video material showing the main functions of the MORPHEUS program.

## Abbreviations

BPD: borderline personality disorder
CIMH: Central Institute of Mental Health
DBT: dialectical behavior therapy
DSM-IV-TR: Diagnostic and Statistical Manual of Mental Disorders, Fourth Edition, Text Revision
DSS-4: Dissociation Tension Scale-4
EMDR: eye movement desensitization and reprocessing
GAD: generalized anxiety disorder
OCD: obsessive-compulsive disorder
PE: Prolonged Exposure
PTSD: posttraumatic stress disorder
SEM: standard error of the mean
STAIR: Skills Training in Affective and Interpersonal Regulation
SUD: subjective units of distress
